# Plasma exchange for treatment of a therapy‐related thrombotic microangiopathy in a patient with advanced hepatocellular carcinoma—A case report

**DOI:** 10.1002/ccr3.8124

**Published:** 2023-11-09

**Authors:** Marco Stortz, Kateryna Shmanko, Daniel Kraus, Simon Gairing, Simone Boedecker‐Lips, Friederich Förster, Arndt Weinmann, Julia Weinmann‐Menke

**Affiliations:** ^1^ Department of Nephrology, Department for Internal Medicine University Medical Center Mainz Mainz Germany; ^2^ Department for Internal Medicine University Medical Center Mainz Mainz Germany; ^3^ Department for Internal Medicine and Clinical Registry Unit University Medical Center Mainz Mainz Germany

**Keywords:** bevacizumab, HCC, plasma exchange, thrombotic microangiopathy

## Abstract

**Key Clinical Message:**

Thrombotic microangiopathies are a side effect of anti‐VEGF therapies, which are often limited to the kidneys but can also occur systemically and be life‐threatening. Screening for increasing proteinuria is essential.

**Abstract:**

We present the case of a 65‐year‐old male patient with a multifocal HCC, Barcelona clinic liver cancer (BCLC) classification B at the time of diagnosis. The HCC was treated with nine sessions of transarterial chemoembolization (TACE), and after a progress, the therapy was switched to a combination of atezolizumab and bevacizumab. Five months after therapy change, he presented with an acute kidney injury. The histopathology of the renal biopsy showed findings of a thrombotic microangiopathy (TMA), which we treated with 12 sessions of therapeutic plasma exchange in combination with steroids, resulting in a decreased TMA activity and later in a remission of the TMA. This case suggests the importance of monitoring the kidney function and proteinuria in patients under anti‐vascular endothelial growth factor (VEGF) therapy and shows a rare differential diagnosis for a worsening of kidney function in these patients. Furthermore, it shows that therapeutic plasma exchange might be a valuable therapeutic option for patients with TMA due to anti‐VEGF therapy.

## Graphical abstract

Visualization of the clinical course of the patient with therapy‐related TMA over time: AKI (acute kidney injury), HCC (hepatocellular carcinoma), TACE (transarterial chemoembolization), BCLC (Barcelona clinic liver cancer), TPE (therapeutic plasma exchange), and HD (hemodialysis).

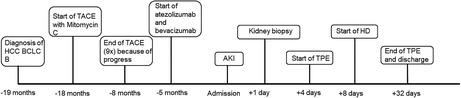

## 1 INTRODUCTION

Drug‐induced thrombotic microangiopathies (DITMA) account for 10%–13% of all thrombotic microangiopathies (TMAs) and contribute to 20%–30% of all secondary TMAs.^1^ Within the group of DITMAs, bevacizumab and mitomycin are most commonly described as the triggering agents.^1^ TMAs can manifest systemically and lead to thrombocytopenia and hemolytic anemia, but they can also have an atypical or subclinical course, especially when confined to an organ, such as renal‐limited TMAs. The majority of renal‐limited TMAs are drug‐induced (28.5%), primarily by anti‐vascular endothelial growth factor (VEGF) substances.^1^ In the vast majority of cases, a DITMA (drug‐induced thrombotic microangiopathy) spontaneously resolves or only requires supportive therapy upon discontinuation of the triggering substance, so there is limited evidence regarding further treatments, like therapeutic plasma exchange (TPE), for DITMA.^1^

We report here the case of a 65‐year‐old patient with renal‐limited TMA during anti‐VEGF therapy, which progressed to a systemic TMA and was successfully treated with therapeutic plasma exchange (TPE).

## 2 CASE REPORT/CASE PRESENTATION

A 65‐year‐old male was referred to our department after external laboratory findings consistent to an acute kidney injury. Four weeks ago, kidney function was in a normal range. At the time of the admission, he complained about lack of appetite, a decline in his general condition, dyspnea, nausea in the morning, and whole‐body pain. The urine output was normal and no dysuria or change in color of the urine. The daily fluid intake was about 1.5 L. The findings in physical examination were normal, except a pressure pain in the right epigastrium and an increased liver size found by examination.

**FIGURE 1:**
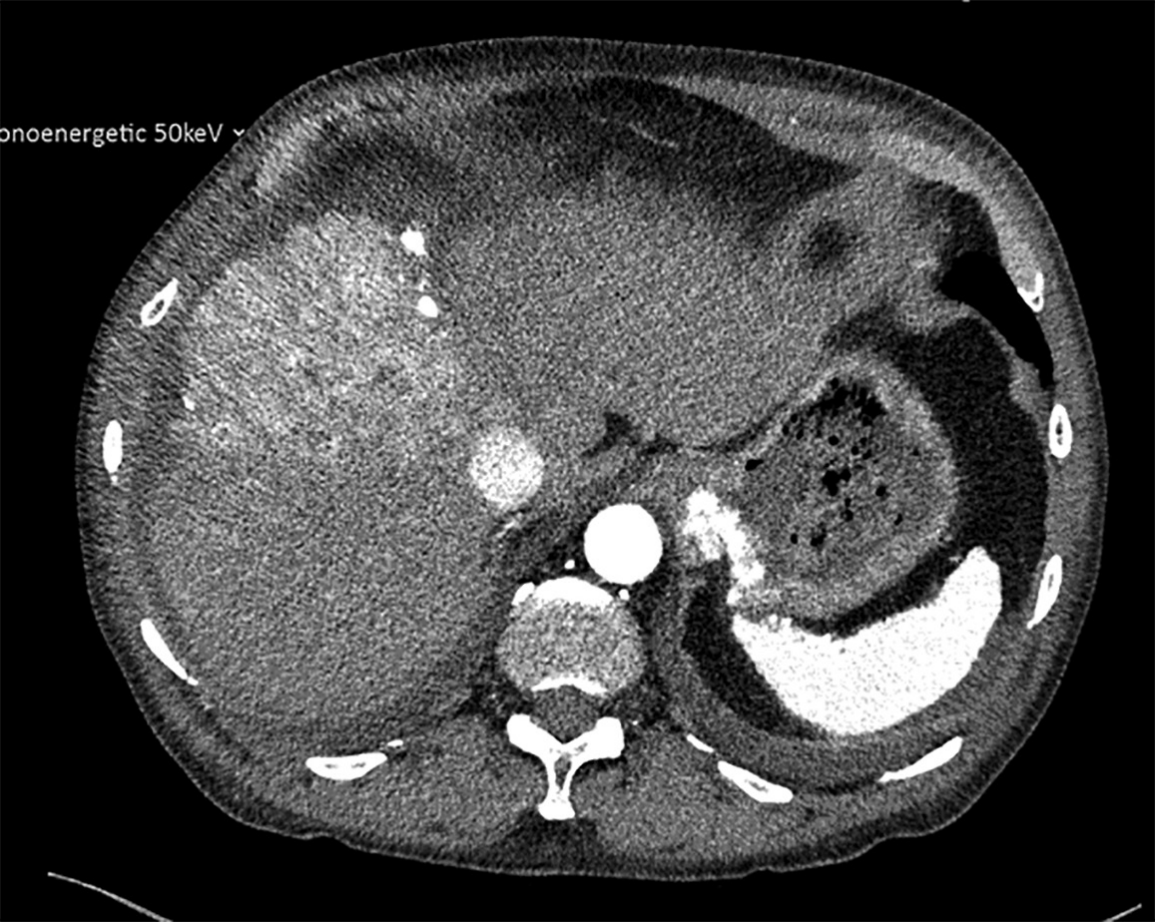
Computed tomography of the upper abdomen at the time of referral to our department: hypervascularized tumor focus in segment VIII/IVa (9 × 9 cm) with washout in the late phase and necrotic arreals.

A multifocal hepatocellular carcinoma due to chronic hepatitis C infection and chronic alcohol abuse but without signs of cirrhosis (BCLC B) had been diagnosed 19 months before in an external clinic. The primary therapy was a TACE with mitomycin, overall, nine times. Therapy by TACE was terminated due to a new tumor manifestation in the liver. Therefore, systemic therapy with atezolizumab and bevacizumab every 3 weeks was started 5 months before the admission to our hospital. Therapy was paused several weeks prior to assignment due to worsening renal function. Overall, the patient received these agents five times. The extent of tumor presentation at the time of initial evaluation is depicted in Figure 1.

Except for an increased creatinine and blood urea nitrogen (BUN), a mild normochromic, normocytic anemia, and a mild albuminuria, laboratory values inclusive autoimmune serology were noticeable. After exclusion of a postrenal genesis of the acute kidney injury and a fluid‐challenge without improvement of kidney function, we performed a kidney biopsy.

**FIGURE 2:**
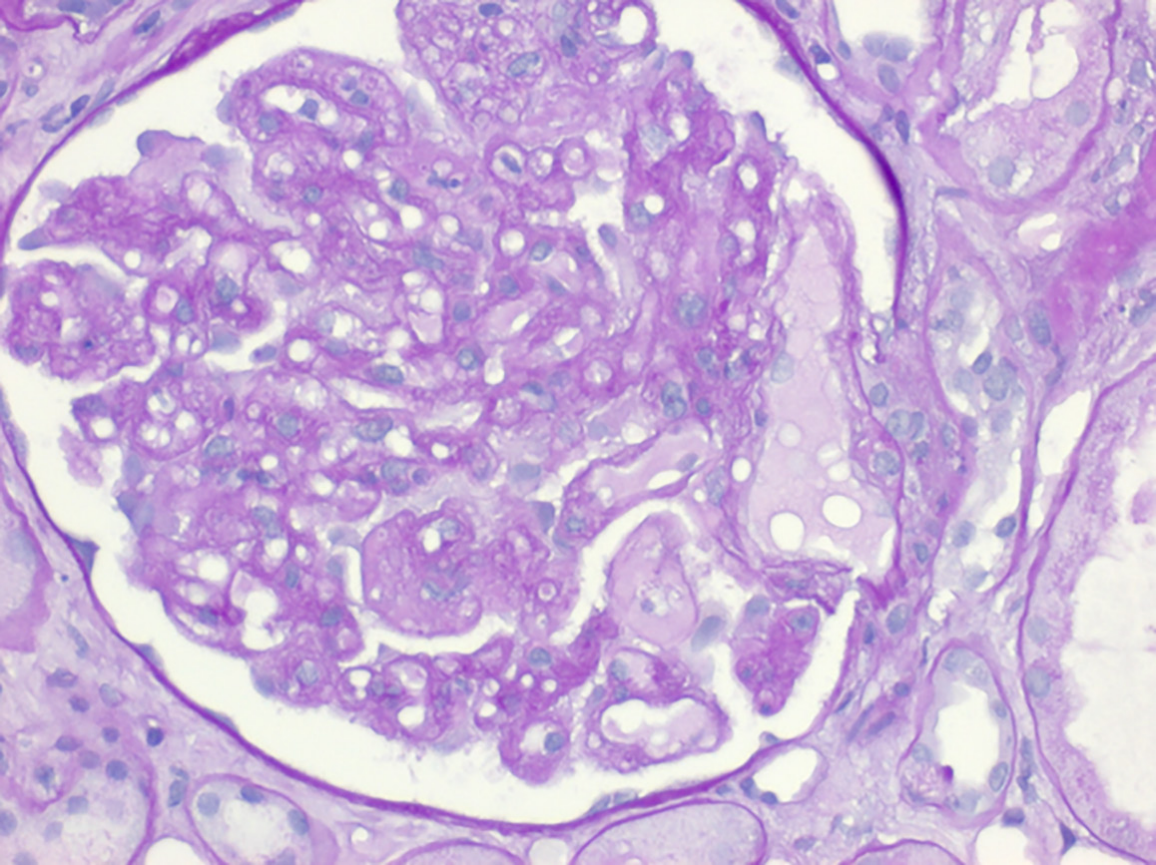
Light microscopic findings (HE staining) with the characteristic findings of anti‐vascular endothelial growth factor therapy‐induced glomerular microangiopathy (aVGF‐GMA): single glomerular microaneurysms and segmental hyalinosis, whereas the preglomerular vascular alterations are untypical for aVGF‐GMA.

The histopathological examination revealed the presence of glomerular capillary microaneurysms and segmental hyalinosis with signs of a severe acute tubular epithelial damage. Some aspects were typically for a TMA referred to bevacizumab treatment but not suitable to these findings, also the preglomerular arterioles were affected with thrombocyte‐thrombi (Figure 2). Since there were no clinical findings suspicious for a typical hemolytic uremic syndrome (HUS), we determined the activity of A disintegrin and metalloprotease with thrombospondin‐1‐like domains (ADAMTS‐13) and the concentration of antibodies against ADAMTS‐13 to exclude another reason for the TMA.

**FIGURE 3:**
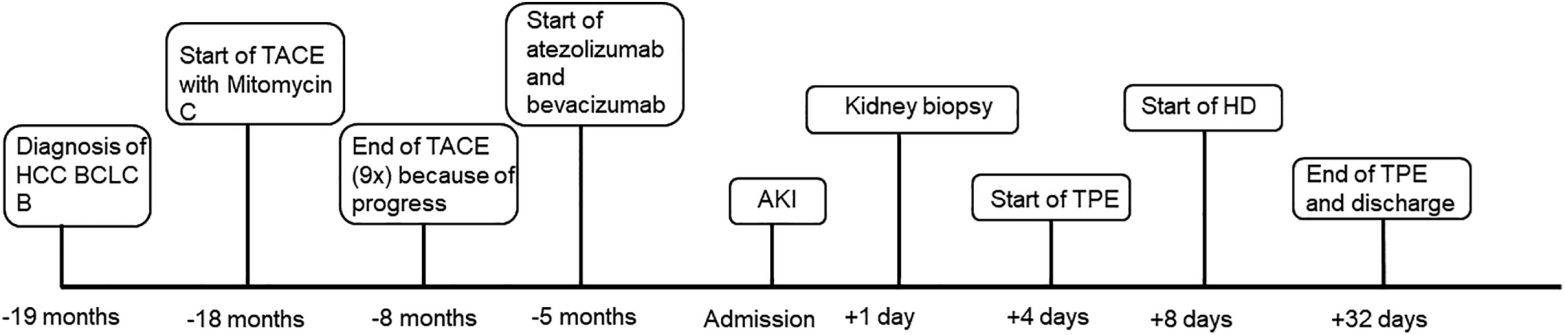
Visualization of the clinical course over time: AKI (acute kidney injury), HCC (hepatocellular carcinoma), TACE (transarterial chemoembolization), BCLC (Barcelona clinic liver cancer), TPE (therapeutic plasma exchange), and HD (hemodialysis).

Because of the dramatic impairment of the kidney function, we started a therapy with high‐dose prednisolone and initiated a plasma exchange therapy with frozen fresh plasma for the purpose of preserving the remaining kidney function. After the third plasma exchange therapy, we saw laboratory findings of a systemic TMA with decreased concentrations of thrombocytes, increased lactate dehydrogenase (LDH) activity, decreased concentration of haptoglobine, and more than 20 schistocytes per 1000 erythrocytes. After the sixth plasma exchange, we started with additional hemodialysis because of symptomatic uremia and signs of hydropic decompensation. After the tenth plasma exchange therapy the thrombocyte concentration increased for the first time and the LDH activity decreased. Nevertheless, we continued plasma exchange therapy for another two times to keep the thrombocyte count stable. An overview of the clinical course is shown in Figure 3.

Today, 1 year after the first plasma exchange, the patient is still undergoing a dialysis treatment three times a week because of the absence of a renal recovery. There is no more activity of the TMA. The treatment regime for HCC has been switched first to sorafenib and after progress under Sorafenib to cabozantinib. In the first staging study after the start of therapy with Cabozantinib, further progression was already seen, so that a renewed therapy switch to nivolumab in combination with ipilimumab, analog to the CheckMate 040‐protocoll, was made. Under this therapy regimen, a reduction in size of most HCC foci occurred.

## 3 DISCUSSION/CONCLUSION

This case report refers to a case of TMA during systemic treatment with the anti‐PD‐L1 antibody Atezolizumab in combination with the anti‐VEGF antibody bevacizumab in a patient with multilocal HCC after pretreatment with conventional transarterial chemoembolization using mitomycin C. To the best of our knowledge, this is the first case report of a patient with HCC and therapy‐induced TMA treated with therapeutic plasma exchange.

Even though renal‐limited TMA is a very rare condition, it is also the most frequent histopathological finding in the kidney under therapy with bevacizumab.^2^ TMA caused by anti‐VEGF agents has a characteristic histopathological morphology and has to be distinguished from TMAs by other causes.^3^ Although it is the most frequent severe adverse event concerning the kidney, TMA is a rare adverse event regarding all patients treated with bevacizumab. In nearly all cases of a bevacizumab‐associated TMA, the microangiopathy is limited to the kidney with no or only mild hematological alterations and ceases after interruption of the medication.^4^ In our case, untypically for bevacizumab, there were also hematological findings of TMA and no spontaneous cessation of TMA activity as well as recovery of renal function after ending therapy, what is the recommended therapy of a DITMA and usually sufficient.^1^ These findings are, in contrast to the histopathological findings, more typical for a TMA caused by mitomycin, which occurs characteristically 6–12 months after drug administration.^4^ TMA caused by mitomycin is typically more severe and occurs generalized with hematological findings and severely compromised kidney function without recovery,^4^, ^5^ as seen in our patient, but it is unusual in cases where mitomycin is administered locally within a TACE. Mitomycin‐induced TMA cannot be ruled out with certainty, as the clinical presentation fits, but we strongly assume an induction of the TMA by VEGF‐inactivation due to bevacizumab, as this assessment is supported by the histopathological findings and temporal context.

We decided to treat our patient with therapeutic plasma exchange in the hope of renal recovery and because of worsening of the renal‐limited TMA to a systemic TMA with hematological abnormalities and continued this therapy. There is very limited evidence of plasma exchange as a therapy in DITMA.^5^, ^6^ In a French registry, only 8 of 1000 patients with TMA after anti‐VEGF therapy underwent a plasma exchange with uncertain outcome.^7^ Data for treatment efficacy of TPE for treatment of DITMAs not antibody mediated are missing.^1^ Good data supporting the favorable clinical outcomes resulting from the use of TPE are available only for some primary TMAs, such as TTP, or for ticlopidine‐induced DITMA, which is antibody mediated.^8^ Nevertheless, TPE is recommended in DITMA therapy in severe cases where drug cessation alone is not successful.^1^ In our described case, the plasma exchange was effective, at least regarding the systemic activity of the drug‐induced TMA and the hematological deteriorations.

Bevacizumab and other agents that interfere into the VEGF pathway by blocking VEGF, its receptors, or the receptor‐linked tyrosine kinase are supposed to inhibit the angiogenesis of tumors.^9^ Practitioners must consider that VEGF is a crucial signal molecule which is produced by podocytes and ensures the integrity of the glomerular basal membrane and the endothelium of the glomerular capillaries.^10^ For this reason, the common side effects of these agents related to the kidney are proteinuria and hypertension.^9^ All patients under therapy with agents interfering with the VEGF pathway should be monitored at least for proteinuria to perform a kidney biopsy in case of a significant increase in amount of proteinuria to detect a TMA in early stages and discontinue therapy to prevent an irreversible renal damage.

## AUTHOR CONTRIBUTIONS

**Marco Stortz:** Conceptualization; writing – original draft. **Kateryna Shmanko:** Resources. **Daniel Kraus:** Supervision; writing – review and editing. **Simon Gairing:** Resources. **Simone Boedecker‐Lips:** Resources. **Friederich Förster:** Resources. **Arndt Weinmann:** Conceptualization; supervision; writing – review and editing. **Julia Weinmann‐Menke:** Conceptualization; project administration; resources; supervision; writing – review and editing.

## FUNDING INFORMATION

This case report was not funded or sponsored.

## CONFLICT OF INTEREST STATEMENT

The authors have no conflicts of interest to declare.

## ETHICS STATEMENT

The present case report complies with the guidelines for human studies and is ethically in accordance with the World Medical Association Declaration of Helsinki.

## CONSENT

The patient which case is presented gave his written informed consent to publish the details of his medical case including the shown images.

## Data Availability

The underlying medical data can be obtained in anonymized form by the corresponding author after written informed consent by the described patient.
